# The role of a probiotics mixture in the treatment of childhood constipation: a pilot study

**DOI:** 10.1186/1475-2891-6-17

**Published:** 2007-08-04

**Authors:** Noor-L-Houda Bekkali, Marloes EJ Bongers, Maartje M Van den Berg, Olivia Liem, Marc A Benninga

**Affiliations:** 1 Department of Paediatric Gastroenterology and Nutrition, Academic Medical Centre, Amsterdam, The Netherlands

## Abstract

**Background:**

Inconsistent data exist about the efficacy of probiotics in the treatment of constipation. Several studies in adults with constipation showed positive effects of probiotics on constipation. Inconsistent data exist regarding the effect of a single probiotic strain in constipated children. The aim of this pilot study was to determine the effect of a mixture of probiotics containing bifidobacteria and lactobacilli in the treatment of childhood constipation.

**Methods:**

Children aged 4–16 years with constipation as defined by the Rome III criteria were eligible for the study. During a 4 week period, children received a daily mix of 4 × 10^9 ^colony forming units of a probiotic mixture (*Ecologic*^®^*Relief*) containing Bifidobacteria (B.) bifidum, B. infantis, B. longum, Lactobacilli (L.) casei, L. plantarum and L. rhamnosus. Primary outcome measures were frequency of bowel movements (BMs) per week and stool consistency. Secondary outcome measures were number of faecal incontinence episodes per week, abdominal pain and side effects.

**Results:**

Twenty children, 50% male, median age 8 (range 4–16) were included.

The frequency of BMs per week increased from 2.0 (1.0–5.0) to 4.2 (0.0–16.0) in week 2 (p = 0.10) and 3.8 (2.1–7.0) in week 4 (p = 0.13). In 12 children presenting with <3 BMs/week, BMs per week increased significantly from 1.0 (0.0–2.0) to 3.0 (0.0–7.0) in week 2 (p = 0.01) and 3.0 (0.0–10.0) in week 4 (p = 0.01). The stool consistency was reported as hard in 7 children at baseline, in 4 children at week 2 (p = 0.23) and in 6 children after 4 weeks of treatment (p = 1.00). A significant decrease in number of faecal incontinence episodes per week was found in the entire group: 4.0 (0.0–35.0) to 1.5 (0.0–14.0) in week 2 (p = 0.01) and 0.3 (0.0–7.0) in week 4 (p = 0.001). The presence of abdominal pain decreased significantly from 45% to 25% in week 2 (p = 0.04) and 20% at week 4 (p = 0.006). No side effects were reported.

**Conclusion:**

This pilot study shows that a mixture of probiotics, has positive effects on symptoms of constipation. To confirm these findings, a large randomised placebo controlled trial is required.

## Background

Functional constipation is a common and frustrating phenomenon in children. The prevalence of childhood constipation in the western world is 1–30% [1]. No organic cause is found in 90% to 95% of those constipated children [2]. This functional defecation disorder is characterized by infrequent defecation less than three times per week, more than two episodes of faecal incontinence per week, the passage of large and painful stools which clog the toilet and retentive posturing. Upon physical examination a palpable faecal mass is often found in the abdomen and the rectum [3,4].

Childhood constipation is usually treated with a combination of toilet training, a bowel diary and oral laxatives such as lactulose or polyethylene glycol (PEG). Laxatives aim to soften the stools, thereby contributing to a break-through of the vicious circle of defecation avoidance caused by pain during defecation. Only 60% of constipated children accomplish successful treatment with laxatives [5]. It is clear that development of other treatment options is required.

There is growing interest in the use of probiotics in organic and functional gastrointestinal disorders. Probiotics are live microbial food ingredients which are reported to be effective in the treatment of IBD, travellers diarrhoea and constipation [6-9]. Colonic microflora influences the peristalsis of the colon [10]. Therefore, imbalance in the colonic microflora has been suggested to play a role in gastro-intestinal diseases such as constipation. Probiotics, such as Bifidobacteria (B.) and lactobacilli (L.), both produce lactic, acetic and other acids resulting in a lowering of pH in the colon. A lower pH enhances peristalsis of the colon and subsequently decreases colonic transit time which is beneficial in the treatment of constipation [10,11]. The latter hypothesis was confirmed showing a decrease in colonic transit time in healthy adults consuming *B. animalis *as supplement [12].

To date, several studies have been performed, mainly in adults, in order to determine the effects of probiotics on symptoms of constipation [10-15]. It has been shown that probiotic strains, such as *L. shirota *and the *B. infantis*, increase defecation frequency and soften stools in adults with constipation and IBS [13,16]. A recent study in children with constipation showed an increase in defecation frequency and a decrease in abdominal pain using the strain *L. rhamnosus *[9]. In contrast to the latter study however, the probiotic strain *Lactobacillus GG *did not have an additional positive effect on constipation symptoms, when used as an adjunctive therapy with lactulose [14].

In constipated elderly, two different strains the Lactobacillus rhamnosus and Propionibacterium freudenreichii resulted in a small but significant increase in defecation frequency, whereas the use of a single strain did not affect defecation frequency [17].

The interpretation of these clinical trials is difficult to compare due to the differences in endpoints, variations in probiotics used, dose and strains. Nonetheless, a recent review suggested that overall, sufficient evidence is available to warrant further evaluation [18].

Therefore we hypothesized that a combination of several strains of bifidobacteria and lactobacilli might be effective in the treatment of childhood constipation. In a pilot study, we aimed to determine the therapeutic effect of a combination of probiotics strains, containing the bifidobacteria *B. bifidus*, *B*. *infantis *and *B. longum and the lactobacilli L. casei, L. plantarum *and *L. rhamnosus*, on childhood constipation.

## Methods

### Subjects

Children between 4 to 16 years of age referred to the outpatient clinic of the Emma Children's Hospital in Amsterdam, the Netherlands, with constipation were eligible for study entry. Childhood constipation was defined by the Rome III criteria as having at least 2 out of 6 of the following symptoms: bowel movements <3 times/week; faecal incontinence >2 times/week; large amounts of stools obstructing the toilet once in 10 days; painful defecation; withholding behaviour; palpable abdominal or rectal mass on physical examination [4]. Exclusion criteria were the use of any oral laxative < 4 weeks before intake, mental retardation, metabolic disease, functional non-retentive incontinence, and a history of gastro-intestinal surgery. All children older than 12 years and/or parents gave informed consent. This pilot was approved by the medical ethical committee of the Academic Medical Centre of Amsterdam.

### Study design

Seven days prior to baseline assessment and during the treatment period all children recorded frequency of bowel movements, the number of faecal incontinence episodes, stool consistency, abdominal pain, flatulence and pain during defecation as well as adverse effects such as vomiting and diarrhoea in a standardized bowel diary.

At baseline assessment, a medical history and information on the current defection pattern was collected. Additionally, a physical examination including a rectal digital exam was performed to assess whether an abdominal or rectal faecal mass was present.

Before start of the probiotics treatment, all children received once daily for 3 days a rectal enema (Klyx: sodium-dioctylsulfosuccinate and sorbitol) in order to accomplish rectal disimpaction. After rectal disimpaction, children were administrated a daily probiotics mixture of 4 × 10^9 ^colony forming units (CFU), containing *Bifidobacteria (B.) bifidum, B. infantis, B. longum, Lactobacilli (L.) casei, L. plantarum *and *L. rhamnosus *(*Ecologic*^®^*Relief, Winclove Bio Industries BV, The Netherlands*) for 4 weeks. During the treatment period children were instructed to start toilet training. Toilet training consisted of sitting on the toilet 3 times per day for 5 minutes after each meal with the intention of trying to defecate. The use of laxatives was not allowed during the short treatment period.

Evaluation was conducted during visits to the outpatient clinic at 2 and 4 weeks after start of treatment. During each visit the physician assessed the patient's daily bowel diary and examined the child.

### Outcome measures

Primary outcome measures were frequency of bowel movements per week and stool consistency. Stool consistency was rated by the patients as hard, normal or watery. Secondary outcome measures were number of faecal incontinence episodes per week, presence of abdominal pain and incidence of adverse effects such as vomiting and diarrhoea.

### Analysis

Descriptive statistical measures were calculated for baseline characteristics using SPSS version 12.0.1 statistical software (SPSS Inc, Chicago, Ill). Change of frequency of bowel movements and faecal incontinence was assessed using the non-parametric paired Wilcoxon test. For the analysis of change of stool consistency, the Mc Nemar test was used. For the comparison of abdominal pain between baseline and the evaluation time points, the Wilcoxon rank test was used. All continuous values were expressed as median (range). A p- value < 0.05 was considered significant.

## Results

Between February 2006 and July 2006, 20 children were enrolled into this pilot study and all patients completed the study. Baseline characteristics are summarized in table 1. In 85% of the children, onset of constipation symptoms was between 0 to 4 years of age.

**Table 1:** Baseline characteristics

**Baseline characteristics**	
**Number of subjects**	20
**Age in years**	8 (4–16)
**Sex (male)**	10
**Time of constipation before intake (years)**	3.5 (0.3–6.5)
**Treatment time before intake (months)**	12 (0–48)

**Bowel habits, n (%)**	
• Bowel movements < 3/week	12 (60%)
• Faecal incontinence ≤ 2/week	1 (5%)
• Faecal incontinence > 2/week	15 (80%)
• Large amounts of stools	12 (60%)

**Stool consistency, n (%)**	
• Hard stools	7 (35%)
• Normal stools	13 (65%)
• Watery stools	0

**Painful defecation, n (%)**	
• No pain	7 (35%)
• Sometimes painful	7 (35%)
• Always painful	6 (30%)

**Abdominal pain, n (%)**	
• No abdominal pain	3 (15%)
• Sometimes abdominal pain	8 (40%)
• Often abdominal pain	9 (45%)

**Physical examination, n (%)**	
• Abdominal scybala	4 (20%)
• Rectal scybala	4 (20%)
• Anal Fissures	0

**Figure 1:**
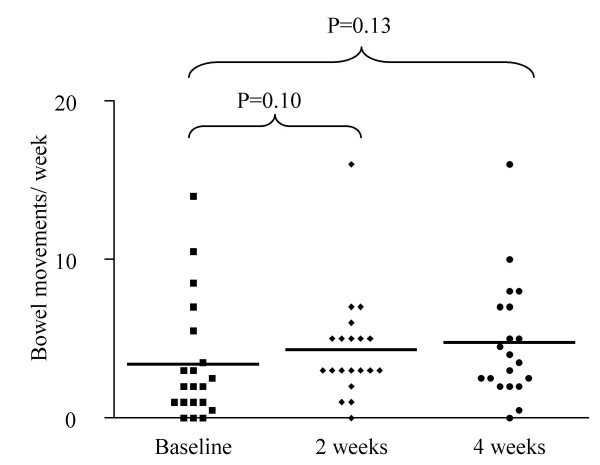
Bowel movements per week in all children.

**Figure 2:**
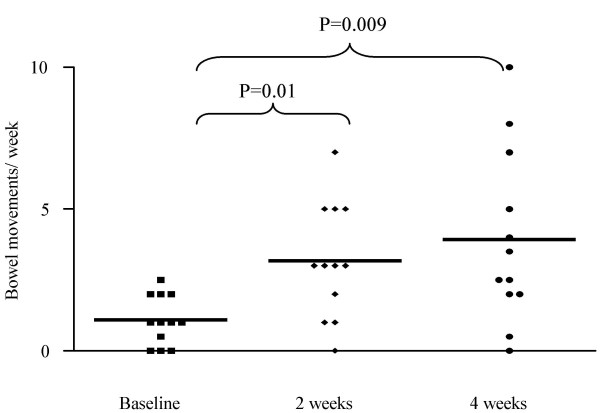
Bowel movements (BMs) per week in children presenting with <3 BMs/week.

The frequency of bowel movements (BMs) per week increased from 2.0 (1.0–5.0) to 4.2 (0.0–16.0) in week 2 (p = 0.10) and 3.8 (2.1–7.0) in week 4 (p = 0.13) (figure 1). In 12 children presenting with <3 BMs per week, BMs per week increased significantly from 1.0 (0.0–2.0) to 3.0 (0.0–7.0) in week 2 (p = 0.01) and 3.0 (0.0–10.0) in week 4 (p = 0.009) (figure 2).

The stool consistency was reported as hard in 7 children at baseline, in 4 children at week 2 (p = 0.23) and in 6 children at week 4 (p = 1.00). At week 4, hard stools appeared in 5 children who also had hard stools at baseline. One child with normal stools at baseline, reported hard stools only at the end of the study. Two of the 7 children who presented with hard stools, reported normal stools at the end of the study.

**Figure 3:**
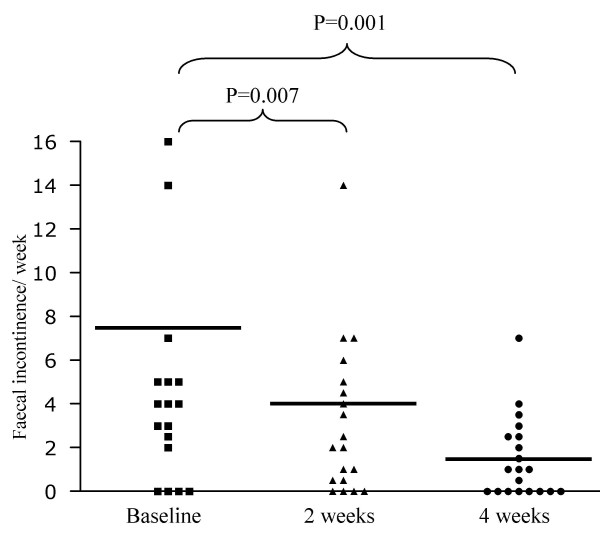
Number of faecal incontinence episodes per week in all children.

The number of faecal incontinence episodes per week decreased significantly from 4.0 (0.0–35.0) to 1.5 (0.0–14.0) in week 2 (p = 0.007) and 0.3 (0.0–7.0) in week 4 (p = 0.001) (figure 3).

The presence of abdominal pain decreased significantly from 45% (n = 9) to 25% (n = 5) in week 2 (p = 0.04) and 20% (n = 4) at week 4 (p = 0.006). There were no side effects such as vomiting, bloating and increased flatulence during the study period.

## Discussion

This pilot study showed that a probiotics mixture containing different strains of bifidobacteria and lactobacilli, increases the frequency of bowel movements in constipated children presenting with a defecation frequency of less than 3 times per week. This probiotic mixture was also effective in decreasing the number of faecal incontinence episodes and in reducing the presence of abdominal pain. No significant changes in stool consistency were found.

Given their safety profile, probiotics could be an attractive compound to manipulate gastrointestinal motility in constipated children [19]. However, exact mechanisms underlying enhancement of gastrointestinal transit are not yet unravelled. Based on the results of our pilot study we hypothesise that a mixture of bifidobacteria and lactobacilli producing lactic, acetic and other acids resulting in a lowering of pH in the colon are effective in enhancing motility of the colon, subsequently leading to a decrease in colonic transit time. A large randomized placebo controlled trial is necessary to confirm these findings.

In this study, we found that administration of a mixture of probiotics had a positive effect on frequency of bowel movements and consequently leading to a decrease in faecal incontinence episodes. In contrast to our findings, Banaszkiewicz showed no additional effect of *lactobacillus GG *(*LGG*) to placebo in children with constipation who were all treated with lactulose. The authors suggested that the probiotic strain LGG may not provide clinical benefits in the treatment of constipation. Furthermore they assumed that the failure of LGG to provide synergistic effects (with lactulose) occurred despite the tempting notion that the concurrent use of lactulose with proven probiotic properties (ie, it promotes growth of lactobacilli in the colon) should enhance the therapeutic effects of LGG. A second study in which a group receives LGG alone, is needed to more directly examine this issue.

It has been assumed that probiotics soften the stools by stimulating water and electrolyte secretion [20-22]. However, we were not able to show a significant softening of stools after 4 weeks of treatment. As only a minority of children (35%) had hard stools at baseline, it is necessary to investigate whether this probiotic mixture has a positive effect on hard stools in a larger randomised controlled trial.

A significant decrease in abdominal pain was found after 4 weeks of treatment with the probiotics mixture. This is in accordance with one paediatric study and several adult studies performed in irritable bowel syndrome (IBS) patients with abdominal pain/discomfort, distension/bloating and difficult defecation [9,23,24]. A recent randomized placebo controlled trial conducted in 360 women with IBS showed that the strain *B. infantis *was associated with significant improvement of both abdominal pain and the subjects' global assessment of symptoms [16]. This positive effect on abdominal pain occurred irrespective of any effect on stool frequency which indicates that the observed effect was not attributable to either a laxative or anti-diarrhoeal effect. It has been suggested previously that abdominal pain and bloating may decrease as a consequence act of probiotics diminishing visceral hypersensitivity by its anti-inflammatory effect on the enteric mucosa [25].

No side effects of the probiotics were found in our study. This is in accordance with literature about the safety of probiotics [19,26]. The safe use of especially bifidobacteria is supported by the long historical consumption of fermented milk and growing knowledge about bifidobacteria taxonomy and physiology. Furthermore, studies performed with lactobacilli and bifidobacteria showed to be well tolerated in adults and children [6,9,14], [26-28].

The interpretation of clinical trials of probiotic strains in functional gastrointestinal disorders is complicated by several factors. Results between studies are difficult to compare due to differences in endpoints, variations in probiotics dose and strains. Whereas one group uses mixtures of probiotics, others use single isolates, making it difficult to determine what were the active moieties [29,30]. Nonetheless, a recent review suggested that overall, sufficient evidence is available to warrant further evaluation [18].

In conclusion, this non randomized non placebo controlled pilot study evaluating the effect of a mixture of probiotics, showed beneficial effects on symptoms of constipation and a decrease of abdominal pain. Therefore a randomised placebo controlled trial is now required to confirm these data.

## Authors' contributions

MBO, MvB and OL contributed substantial to conception and design of the study and collection of the data. MBE supervised the design and coordination of the study and helped to draft and revise the manuscript. All authors read and approved the final manuscript.
